# Isolation of Sulfur Reducing and Oxidizing Bacteria Found in Contaminated Drywall

**DOI:** 10.3390/ijms11020647

**Published:** 2010-02-05

**Authors:** Dennis G. Hooper, John Shane, David C. Straus, Kaye H. Kilburn, Vincent Bolton, John S. Sutton, Frederick T. Guilford

**Affiliations:** 1 RealTime Laboratories, LLC, 13016 Bee Street #203, Dallas, TX 79234, USA; E-Mails: vincebolton@yahoo.com (V.B.); drg@readisorb.com (F.T.G.); 2 PRO-LAB, Weston, FL 33326, USA; E-Mail: jshane@core.com; 3 Department of Microbiology and Immunology, Texas Tech University Health Sciences Center, Lubbock, TX 79430, USA; E-Mail: david.straus@ttuhsc.edu; 4 Neuro-Test, Inc., Pasadena, CA 91107, USA; E-Mail: khkilburn@sbcglobal.net; 5 Embark Scientific, LLC, Austin, TX 78757, USA; E-Mail: ssutton@embarkscientific.com

**Keywords:** Chinese drywall, *Thiobacillus ferrooxidans*, sulfur reducing bacteria, sulfur emissions

## Abstract

Drywall from China has been reported to release sulfur producing products which are corrosive to metals, result in noxious odors, and represent a significant health risk. It has been reported that these emissions produce medical symptoms such as respiratory or asthma type problems, sinusitis, gastrointestinal disorders, and vision problems in home owners and their household pets. We report here a method of identifying a causative agent for these emissions by sampling affected gypsum wallboard and subjecting those samples to Real Time Polymerase Chain Reaction [RT-PCR] studies. Specific DNA probes and primers have been designed and patented that detect a specific iron and sulfur reducing bacterium (*i.e.*, *Thiobacillus ferrooxidans)*. One hundred percent of affected drywall samples obtained from homes located in the southeastern United States tested positive for the presence of *T. ferrooxidans*. All negative controls consisting of unaffected wallboard and internal controls, *Geotrichum* sp., tested negative within our limits of detection.

## 1. Introduction

Corrosive imported drywall refers to drywall imported into the United States from China from 2004 to 2007 [1]. The U.S. Consumer Product Safety Commission has recently received more than 550 reports from people in 19 states and the District of Columbia involving odors, health symptoms and corrosion problems they blame on imported Chinese drywall. The complaints also describe “rotten egg” and “burnt match” smells, corrosion of wiring, and corrosion and/or tarnishing of other metals in the homes [2]. It is reported that the imported drywall emits gases that produce a sulfurous odor which cause significant property damage in heating, ventilation, and air conditioning systems, electrical wiring, copper plumbing, and appliances. The inhabitants of these same buildings report a variety of health related problems [3–5]. The source and/or cause of the property damage has not been identified. There are, however, many who conjecture that the gases arise from tainted drywall manufactured in China which is both the source and cause of both the reported environmental and health problems. One theory is that the tainted drywall was manufactured with gypsum mined in China and that the gypsum was then mixed with fly ash, a waste material that is a byproduct from power plants using coal. Samples of Chinese drywall that were tested by United Engineering, however, found that the wallboard consisted of 5–15% organic material, which contradicts the theory that Chinese drywall was made using waste from coal fired power plants, and indicates that the tainted drywall from China comes from mined gypsum, not synthetic gypsum made from coal ash [5,6]. Since this wallboard contains such a high concentration of organic material, a current theory in the industry is that Chinese drywall contains an organism that is degrading iron and sulfur compounds to produce sulfur odors [7]. The organisms in this study produce hydrogen sulfide and sulfur dioxide which have been documented to cause many symptoms when humans are exposed to them [8,9]. Sulfur reducing bacteria (SRB) make hydrogen sulfide and have recently been linked as a causative agent in disorders of the gastrointestinal tract [10], eyes [11], and respiratory system [12] in humans and animals. Furthermore, testimony before the United States Senate by the Director of the Division of Environmental Hazards and Health Effects emphasized the existing health and product issues associated with imported drywall [13].

Our objective was to determine if sulfur and iron reducing/oxidizing bacteria could be isolated from contaminated wallboard. The organisms studied were: *Thiobacillus(Acidithiobacillus)ferrooxidans, Sulfobacillus thermosulfidooxidans, Leptosprillium ferroo xidans, Thiobacillus (Acidith iobacillus) caldus,* and *Actinobacillus thiooxidans*. All work was conducted on either American Type Culture Collection (ATCC), New York, NY, cultures or DNA obtained directly from ATCC. RealTime polymerase chain reaction studies (RT-PCR] were conducted on the five organisms. The isolation of sulfur reducing/oxidizing bacteria in drywall may explain the reported patient symptoms.

## 2. Results and Discussion

**Figure 1.**
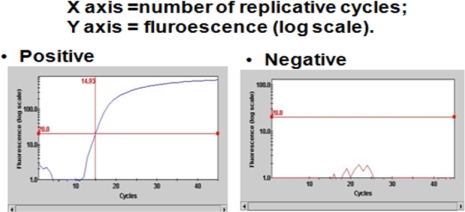
Representative control curves for bacteria tested in contaminated drywall samples.

**Figure 2.**
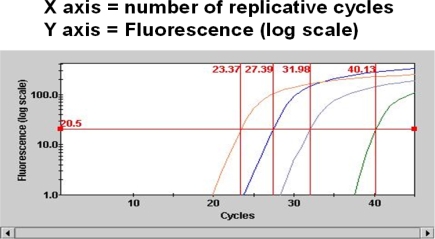
Representative *geotricum* sp. (GEO_control for all tests conducted (internal control). Different concentrations of GEO were added to the same amount of drywall material.

Samples were extracted and subjected to DNA probes and primers to SRB. (See Table 1 for organisms tested). Controls consisting of known positive organisms as well as known negative organisms were used in each test. *Geotrichum* (GEO) was used as an internal control to indicate that the entire system can and does exhibit polymerase chain reaction (PCR) activity. PCR control curves for known positive SRB organisms and known SRB negative (*i.e.*, *Escherichia coli)* as well as the GEO internal control are shown in Figures 1 and 2.

Assays for all organisms were optimized utilizing target DNA in 10-fold serial dilutions formulating multiple curves. Organisms used in this study are shown in Table 1. Data collected and evaluated, as well as the assays were determined to be optimized to the specification of RealTime Laboratories, LLC, Dallas, Texas. The results of the 1:10 dilutions made of each organism tested in water demonstrated the sensitivity of system. Thus, standard PCR curves could be drawn for each organism. (Table 2). These studies were conducted using non-contaminated drywall to show that the components in drywall do not inhibit the PCR reaction. Although the assay validation was not quantitative, the assay was sensitive to five copies of DNA for the organism(s) tested. Samples were considered positive when the replication curve crossed the 20.0 fluorescence mark (log scale). Negative samples did not cross the 20.0 fluorescence mark.

Normal drywall samples (not emitting any sulfur compounds) were purchased from a local building supply store and then tested by spiking them with known ATCC organisms. Normal spiked drywall was tested using each of five DNA probes to the organisms listed in Table 2. A positive GEO control and negative drywall sample was also tested. In all cases of the spiking test, the DNA from all five organisms and the GEO control was detected (Table 2). Drywall samples from homes with known contaminated drywall were then assayed. Assays using probes for all organisms in this study were conducted using all five organisms noted in Table 1. *Thiobacillus ferroxidans* was detected in all twenty-five samples tested. Only one very weak positive result with *T. caldus* was detected in one of twenty-five drywalls tested.

In this study, five organisms were examined to see if they were present in contaminated drywall. The iron-oxidizing bacterium *T. ferrooxidans* is an important organism for the leaching of sulfide ores [14,15]. *Thiobacillus ferrooxidans* can oxidize both Fe^2 +^ and reduced sulfur compounds [16]. Two enzymes have been isolated from *T. ferrooxidans* which can use Fe^3+^ as an electron acceptor for the oxidation of sulfide and sulfite ions. These enzymes, hydrogen sulfide: ferric ion oxidoreductase (SFORase] and sulfite: ferric ion oxidoreductase, have been purified from *T. ferrooxidans. A* new route for sulfur oxidation in *T. ferrooxidans* was reported by Sugio *et al.* in 1989 [17]. They showed that the substrate for SFORase in solid elemental sulfur oxidation by *T. ferrooxidans* was hydrogen sulfide. This system involves a hydrogen sulfide-binding protein (SBP), which reversibly binds hydrogen sulfide and supplies SFORase with hydrogen sulfide as a substrate. Thus, the existence of SBP demonstrated that hydrogen sulfide was synthesized in T*. ferrooxidans* cells. This study concluded that hydrogen sulfide plays a role as an energy reserve in *T. ferroxoidans*. Sugio *et al.* in 1992 [18] and again in 2000 [19] substantiated the existence of a hydrogen sulfide:ferric ion oxidoreductase in iron-oxidizing bacteria such as *T. ferrooxidans*.

Although the source has not yet been identified, analytical testing of contaminated Chinese drywall samples has revealed strontium sulfide, hydrogen sulfide, and sulfur dioxide (SO_2_) [20]. Emissions from the drywall have been blamed for the reported corrosion of plumbing and electrical systems and homeowners have blamed the gases for their health ailments [1–3]. After a report from the Florida Department of Health on October 15, 2009 [3] many questions remained concerning the adverse health effects of the contaminated drywall. Although sulfur gases were found, there was no evidence of high emissions in any of the samples evaluated [21]. These results do not explain the human ailments due to this exposure. Dose response relationships for hydrogen sulfide in human subjects cannot be developed directly because of known adverse effects on function of the brain, eye, lung, and skin [22]. Kilburn showed that the best data have been assembled from observed episodes rather than from experimental exposures [9]. These data showed impairment of balance, color discrimination, and reaction time, verbal memory and other functions at average daily exposures to H_2_S of 1 part per million (ppm) to 5 ppm. There is also evidence that brief spikes of concentrations of H_2_S for 1 to 3 breaths at 50 ppm to 200 ppm. cause coma, while average levels of 100 ppm and higher concentrations are fatal. Also, levels that corrode metal cause brain damage, corneal ulcers, and asthma in human subjects [23,24]. These effects on the brain appeared permanent, judged from studies a year later [9,22] while, in contrast, asthma and corneal ulcers improved.

## 3. Experimental Section

### 3.1. The Hypothesis in This Study Tested the Following:

#### 3.1.1. Null Hypothesis

There was not a significant difference in the presence of sulfur and iron reducing/oxidizing bacteria between contaminated and non-contaminated drywall.

#### 3.1.2. Alternative Hypothesis

There was a significant difference in the presence of sulfur and iron reducing/oxidizing bacteria between contaminated and non-contaminated drywall.

### 3.2. Samples

Samples of contaminated drywall previously tested for the presence of strontium, carbon disulfide, carbonyl sulfide, dimethyl disulfide, and dimethyl sulfide in 25 homes in southeastern US were examined in this study. All tested positive for volatile sulfides. Sulfides are known to be strong oxidants and odorants. In the presence of hydrogen sulfide, carbonyl sulfide will turn copper and silver black and further corrode these metals. All samples were obtained from PRO-LAB, Weston, FL, and were subjected to multiple toxicology, mycology and microbiology examinations. All standard conventional bacterial and mycology cultures were negative. Thus, all studies were non-contributory to causation of the production of hydrogen sulfide and sulfur dioxide. A two square inch piece of dry wall was submitted under chain of custody to RealTime Laboratories. Each specimen was placed in a clean, sterile crucible and crushed into dry powder. One gram of powdered dry wall was then subjected to extraction.

Samples of US manufactured dry wall, not exuding H_2_S and SO_2_ were purchased from a local building supply store and sampled in the same matter as the contaminated dry wall sections. Positive controls were non-contaminated drywall spiked with various concentrations of the five different bacterial organisms examined (Table 1). Internal controls consisted of a known fungal organism, *Geotricum* sp., (GEO Control) which is present to insure that that extraction system is working properly.

### 3.3. Extraction of Samples

Positive and negative controls and all samples were extracted using a commercially available extraction system (Qiagen, Inc., Valencia, CA) that was optimized for the extraction of bacterial genomic DNA. Powdered samples were extracted by pretreatment with Proteinase K and lysis solution in the presence of silica beads for bead beating (Sigma, St. Louis, MO). A set volume of internal control GEO spores was introduced to all samples, including the negative control. All samples were incubated at 55 °C for 1 hour, then at 72 °C for 15 minutes, and then finally further subjected to a second lysis solution. After ethanol addition, the samples were placed into spin columns containing a silica filter. DNA in the mixture was bound to the silica filter, washed several times to remove contaminants and eluted from the spin column filter by utilizing a low salt solution. Samples were stored at −20 °C until tested.

### 3.4. Target Design

Primers and probes specific for each of 5 bacterial targets plus an internal control assay were designed by RealTime Labs, LLC and synthesized by Sigma-Aldrich (St. Louis, MO). All probes used for the assays are hydrolysis probes with the reporter FAM attached to the 5’ end and the quencher Black Hole Quencher (BHQ) attached to the 3’ end. Primers and probes were received lyophilized and re-suspended and mixed into working stocks containing probe and primer at required concentrations. The enzyme for the reaction was purchased as a lyophilized bead from Cepheid (Palo Alto, CA) and manufactured specifically for the SmartCycler® system. Each 50.0 μL SmartCycler® reaction tube contained reaction mix in the formula: 1.0 bead, 33.0 μL of PCR grade water, 7.0 μL primer/probe working stock, and 5.0 μL of extracted DNA. The internal control was run for each extracted sample and a negative control was added as an independent assay to insure a quality control step for the extraction. All 5 bacterial assays and the internal control had a cycling profile containing an initial hot start followed by 45 denature and anneal cycles. All assays were designed and operated as qualitative detection assays. The capacity of a single SmartCycler® instrument varied from 16 to 96 reactions depending on the configuration. Multiple bacterial assays could be processed on a single SmartCycler® run with each assay containing samples, a negative control and a positive control. Data generated from the assays were analyzed utilizing the SmartCycler® Software and positives were defined as any target that crossed the Cepheid recommended threshold of 20 on the fluorescence scale during processing on the instrument.

### 3.5. Assay Design

All assays were designed using sequence data from the strains listed in Table 1 with the assays and utilizing commercially available assay design software (GenBank). Specificity was checked between the organisms using an assembly program. The probe and primer sequence specificity was checked using the Blast tool available at the National Center for Biotechnology Information (NCBI) website.

### 3.6. Assay Optimization

All assays were optimized by utilizing lyophilized stocks of master mix, and then running standard curves. Further optimization was accomplished by testing spiked samples of drywall and actual drywall unknowns.

#### 3.6.1. Rehydration of Lyophilized Stocks

All lyophilized stocks were rehydrated to 100 μM concentration. Master Mix Stocks were made for each assay by using the following formulation for 700.0 μL of Master Mix Stock: Primer 1–15.0 μL; Primer 2–15.0 μL; Probe–10.0 μL; Water–660.0 μL; giving a total master mix stock of 700.0 μL.

#### 3.6.2. Standard Curves

Assays for organisms were optimized utilizing target DNA in 10 fold serial dilutions formulating a curve. Data were collected and evaluated. The assays were optimized to the specification of RealTime Laboratories.

#### 3.6.3. Testing of Spiked Drywall

Assays for organisms were further tested by spiking known organisms into wallboard that was not contaminated with any organisms. This wallboard was tested for each organism along with a positive control and a negative wallboard sample.

#### 3.6.4. Testing of Drywall Unknowns

Assays for organisms were run against 25 unknown drywall samples. Results are shown in Table 2.

## 4. Conclusions

Contaminated drywall can be defined as drywall that emits hydrogen sulfide and sulfur dioxide gases. The control group in this case consists of drywall that does not emit the same types of gases. Results of the testing in this study indicate that the null hypothesis is rejected. There is sufficient evidence to indicate that there are bacteria that can reduce/oxidize sulfur and iron in contaminated drywall and these bacteria are not present in the non-contaminated drywall. This bacterial group is one that reduces and oxidizes sulfur and iron. The significant organism in contaminated dry wall was determined to be *Thiobacillus ferrooxidans.*

## Figures and Tables

**Table 1.** Bacterial organisms and the strains used to create DNA probes/primers.

***Thiobacillus (Actinobacillus) caldus***	Designed with strains N39-30-02, P5-10, MTH-04, DSM8584, DX-2, J-1, D-2, YN06, S-1, T-1, S-2, D-1, and ATCC51756.
***Sulfobacillus thermosulfidooxidans***	Designed with strains HR-K17-45, DK-J16-45, DK-E8-45, N19-45-01, N19-50-01, G-2, YN22, and ATCC 51911 (*S. disulfidooxidans*).
***Leptospirillum ferrooxidans***	Designed with strains JCM17, C2-6, C21, C2-3, C2-7, C2-2, C2-4, and ATCC 53993 D.
***Actinobacillus thioxidans***	Designed with strains ATCC 19377, and ATCC 55020.
***Thiobacillus (Actinobacillus) ferrooxidans***	Designed with strains ATCC2327OT, ATCC33020, SS6, CC1, BRGM1, LMT1, LMT4, DSM9465, SS4, 84, ATCC19859, TF-49, B9, B20, B5, ML17-48A, and ATCC23270D.

**Table 2.** Drywall test results for validation studies of sulfur and iron reducing and oxidizing bacteria.

**Organism**	**Standard Curves Drawn**	**Spiked Drywall Showing No Inhibition of Curves Number spiked/Number Positive**	**Drywall Unknowns Positive/Total Tested (Percent Positive]**
***Thiobacillus (Actinobacillus) caldus***	Yes	6/6	1 (weak)/25 (4.0%)
***Sulfobacillus thermosulfidoxidans***	Yes	6/6	0/25 (0%)
***Leptospirillum ferrooxidans***	Yes	6/6	0/25 (0%)
***Actinobacillus thioxidans***	Yes	6/6	0/25 (0%)
***Thiobacillus (Actinobacillus) ferrooxidans***	Yes	6/6	25/25(100%)
